# Fiber matrix of assay feedstuffs modulates the effect of fat type on standardized ileal amino acid digestibility in broiler chickens

**DOI:** 10.1016/j.psj.2026.107515

**Published:** 2026-07-28

**Authors:** Sujitha Veeraganti, Olanrewaju Ayinde, Oluyinka A. Olukosi

**Affiliations:** Department of Poultry Science, University of Georgia, Athens, GA, 30602, USA

**Keywords:** Dietary fiber, Fat type, Fat level, Amino acid digestibility, Gut morphology

## Abstract

Experiment 1 investigated the interactivity of fiber feedstuff and fat type on standardized ileal amino acid digestibility (**SIAAD**), the morphology of the digestive organs, cecal content pH, and short-chain fatty acid (**SCFA**). Experiment 2 examined how the dietary inclusion level of lard influences the SIAAD of soybean meal (**SBM**). For Expt. 1, 260 broiler chicks were allocated to 52 metabolism cages (six diets with six replicates), plus two nitrogen-free diets (**NFD**). In Expt. 2, 160 broiler chickens were allocated to 32 metabolism cages (four diets with eight replicates). All birds received a basal corn-SBM diet from d 0 to 16, followed by experimental diets from d 16 to 21. In Expt. 1, the diets were semi-purified diets with corn, wheat, or wheat bran as the sole source of dietary protein, each with either soybean oil or lard as the dietary fat (3 × 2 factorial). In Expt. 2, the diets containing SBM as the sole protein source were formulated with four dietary levels of lard (10-40 g/kg). On d 21, ileal digesta (Expt. 1 and 2), and small intestine sections, gizzard, pancreas, and cecal content (Expt. 1 only) were collected. In Expt. 1, there was a significant feedstuff × fat type (P < 0.05) for SIAAD of most AA. The SIAAD of corn was unaffected, whereas wheat and wheat bran had higher SIAAD values with lard and soybean oil, respectively. Birds fed the wheat bran diets had greater (P < 0.05) relative weights of the small intestine and the gizzard. In Expt. 2, a quadratic increase in SIAAD of all AA as the lard level increased was observed (P < 0.01). In conclusion, the the SIAAD effect of assay diets containing different fat type was dependent on the fiber matrix of the assay feedstuff and the quadratic SIAAD response to inclusion level of lard indicates a dose-dependent effect of lard inclusion on AA digestibility.

## Introduction

In our previous studies, we reported that the type of fat used in the assay diets had only a marginal effect on the basal endogenous amino acid flow (**BEL**) and the standardized ileal amino acid digestibility (**SIAAD**) of corn or soybean meal (**SBM**) (Veeraganti et al., 2026). In the current experiments, we evaluated the effects of fat type on SIAAD of feedstuffs differing in fiber profiles. We also examined how varying levels of fat inclusion affect the SIAAD of SBM. Even though several studies have estimated the SIAAD of feed ingredients (Lemme et al., 2004; Olukosi et al., 2019, 2023), published data evaluating the SIAAD of feedstuffs using different fat types are scarce.

The SIAAD estimates obtained by correcting the apparent values with BEL are preferred in feed formulation because of considerable within-feedstuff variation in amino acid (**AA**) digestibility, particularly in ingredients with low crude protein content (Bryden and Li, 2010). The use of SIAAD estimates for diet formulation helps prevent both excess and deficient AA intake (Bandegan et al., 2010). This is critical because AA plays a key role in achieving expected body weight and feed efficiency in broiler chickens (Alabi and Adedokun, 2025). Therefore, supplying AA according to the birds’ actual physiological requirements is essential to support normal metabolic processes (Ullah et al., 2016).

The high fiber content of certain feedstuffs limits their inclusion in broiler diets due to antinutritional factors (Olukosi et al., 2023). However, studies suggest that selecting feedstuffs with appropriate fiber content has beneficial effects on gut morphology, cecal short-chain fatty acids (**SCFA**), and digestibility (Jha et al., 2019). Thus, accurate estimation of the actual digestible AA profile of feedstuffs is critical, irrespective of their fiber profile. On the other hand, dietary fats from different sources exhibit distinct physical and chemical properties and vary in digestibility (Shoaib et al., 2023). Vegetable oils are rich in unsaturated fatty acids and are easily digested, whereas animal fats are the opposite (Tancharoenrat et al., 2013). It can be reasoned that the type of fat used in assay diets may influence SIAAD if it affects the digestibility of AA in the feedstuff.

Therefore, the objectives of the experiments were: 1) to investigate the interaction between feedstuffs that differ in nutrient profile levels, especially fiber, and dietary fat type on SIAAD; and 2) to determine the effect of fat level on SIAAD in SBM. Additionally, phenotypic observations, including digestive organ morphology, cecal short-chain fatty acid (SCFA) profile, and pH of cecal content, were documented in this study to help contextualize the SIAAD response observations.

## Materials and methods

The Institutional Animal Care and Use Committee at the University of Georgia approved the experimental procedures used in the current study.

### Birds and housing

Two experiments were conducted concurrently using male broiler chicks (by-product breeder chicks). For Expt. 1, a total of 260 birds were assigned to 52 battery cages, with 6 replicates for each of the 6 semi-purified dietary treatments and 8 replicates for the 2 nitrogen-free diets, with 5 birds per cage. For Expt. 2, 160 birds were allocated into 32 metabolism cages, with four dietary treatments, eight replicates per treatment, and five birds per replicate cage. Randomized complete block design was used in both experiments. In both experiments, all the birds were fed a basal corn-soybean meal-based diet formulated to meet all nutritional recommendations for Cobb 500 from d 0 to 16. Birds were allocated to their respective treatments and received experimental diets from d 16 to 21. The lighting and temperature schedule followed the Cobb 500 recommendation.

### Experimental diets

#### Expt. 1

The semi-purified or assay diets were formulated with corn, wheat, or wheat bran as the sole protein source. Within each assay feedstuff, diets containing soybean oil or lard were formulated to be isonitrogenous, allowing the effect of fat type to be evaluated in each feedstuff (Table 1). Two nitrogen-free diets (**NFD**) were formulated using two fat types (soybean oil or lard). The composition of the NFD is presented in Table 2. Basal endogenous amino acid losses obtained from the respective NFD were used to calculate SIAAD values for assay diets containing the corresponding fat type.

**Table 1:** Feedstuffs and chemical compositions (g/kg) of experimental diets from d 16 to d 21 for Expt. 1.

Items	Corn		Wheat		Wheat bran	
Corn	939.4	939.4				
Wheat			934	934		
Wheat bran					908.3	908.3
Soybean oil	15.0		20.0		50.0	
Lard		15.0		20.0		50.0
Dicalcium Phosphate	24.4	24.4	24	24	16.9	16.9
Limestone	7.1	7.1	7.2	7.2	10.4	10.4
Titanium dioxide	3.0	3.0	3.0	3.0	3.0	3.0
Vitamin premix^1^	1.0	1.0	1.0	1.0	1.0	1.0
Trace Mineral premix^2^	0.8	0.8	0.8	0.8	0.8	0.8
Choline chloride	5.0	5.0	5.0	5.0	5.0	5.0
Salt	2.5	2.5	2.5	2.5	2.3	2.3
Sodium bicarbonate	1.8	1.8	2.5	2.5	2.5	2.5
Total	1000	1000	1000	1000	1000	1000
Calculated nutrients and energy, g/kg						
Crude protein	61.1	61.1	99.9	99.9	139	139
ME, kcal/kg	3146	3144	2970	2967	1806	1798
Ca	9.6	9.6	9.6	9.6	9.6	9.6
Total P	7.4	7.4	7.1	7.1	13.8	13.8
Non-phytate P	5.4	5.4	5.4	5.4	5.4	5.4
Digestible amino acids, g/kg						
Arg	3.2	3.2	4.9	4.9	9.0	9.0
His	1.9	1.9	2.3	2.3	3.6	3.6
Ile	2.2	2.2	3.3	3.3	4.5	4.5
Leu	8.2	8.2	6.6	6.6	8.4	8.4
Lys	1.9	1.9	2.9	2.9	5.7	5.7
Met	1.4	1.4	1.6	1.6	2.3	2.3
Cys	1.5	1.5	2.3	2.3	2.8	2.8
Thr	2.3	2.3	2.9	2.9	4.6	4.6
Trp	0.5	0.5	1.3	1.3	2.0	2.0
Val	3.0	3.0	4.2	4.2	6.4	6.4
TSAA	2.9	2.9	3.9	3.9	5.1	5.1
Phe + Tyr	5.5	5.5	6.9	6.9	9.4	9.4

1 Vitamin A, 5484 IU; vitamin D3, 2643 ICU; vitamin E, 11 IU; menadione sodium bisulfate, 4.38 mg; riboflavin, 5.49 mg, d-pantothenic acid, 11 mg; niacin, 44.1 mg, choline chloride, 771 mg; vitamin B12, 13.2 µg; biotin, 55.2 µg; thiamine mononitrate, 2.2 mg; folic acid, 990 µg; pyridoxine hydrochloride, 3.3 mg.

2 Iodine, 1.11 mg; manganese, 66.06 mg; copper, 4.44 mg; iron, 44.1 mg; zinc, 44.1 mg; selenium, 300 µg.

**Table 2:** Feedstuffs and chemical compositions (g/kg) of nitrogen-free diets from d 16 to d 21 for Expt. 1.

Items	NFDS^1^	NFDL^2^
Corn-starch	217.2	217.2
Dextrose	651.6	651.6
Soybean oil	25	
Lard		25
Dicalcium phosphate	26.5	26.5
Limestone	6.3	6.3
Titanium dioxide	3	3
Vitamin premix^3^	1	1
Trace mineral premix^4^	0.8	0.8
KCl	4	4
Mg oxide	0.7	0.7
Choline chloride	5	5
Sodium bicarbonate	9	9
Solka-floc	50	50
Total	1000	1000
Calculated nutrients and energy, g/kg		
Crude protein	0	0
ME, kcal/kg	3508	3504
Ca	9.6	9.6
Total P	5.4	5.4
Non-phytate P	5.4	5.4

1 Nitrogen-free diet with soybean oil.

2 Nitrogen-free diet with lard.

3 Vitamin A, 5484 IU; vitamin D3, 2643 ICU; vitamin E, 11 IU; menadione sodium bisulfate, 4.38 mg; riboflavin, 5.49 mg, d-pantothenic acid, 11 mg; niacin, 44.1 mg, choline chloride, 771 mg; vitamin B12, 13.2 µg; biotin, 55.2 µg; thiamine mononitrate, 2.2 mg; folic acid, 990 µg; pyridoxine hydrochloride, 3.3 mg.

4 Iodine, 1.11 mg; manganese, 66.06 mg; copper, 4.44 mg; iron, 44.1 mg; zinc, 44.1 mg; selenium, 300 µg.

#### Expt. 2

Four isonitrogenous diets were formulated by incorporating increasing levels of lard (10, 20, 30, or 40 g/kg) into soybean meal-based semi-purified diets, yielding the four dietary treatments. For this experiment, BEL values determined from the lard-based NFD from Expt. 1 were applied to all assay diets regardless of lard inclusion level. The ratio of corn-starch to dextrose was held constant across the four diets. The formulas for the experimental diets are in Table 3.

**Table 3:** Feedstuffs and chemical compositions (g/kg) of experimental diets from d 16 to d 21 for Expt. 2.

Items	L10^1^	L20^2^	L30^3^	L40^4^
Soybean meal	326	326	326	326
Corn-starch	154.4	151.9	149.4	146.9
Dextrose	463.3	455.8	448.3	440.8
Lard	10	20	30	40
Dicalcium Phosphate	23.2	23.2	23.2	23.2
Limestone	6.2	6.2	6.2	6.2
Titanium dioxide	5.0	5.0	5.0	5.0
Vitamin premix^5^	1.0	1.0	1.0	1.0
Trace Mineral premix^6^	0.8	0.8	0.8	0.8
Choline chloride	5.0	5.0	5.0	5.0
Salt	2.5	2.5	2.5	2.5
Sodium bicarbonate	2.5	2.5	2.5	2.5
Total	1000	1000	1000	1000
Calculated nutrients and energy (g/kg)				
Crude Protein	150.3	150.3	150.3	150.3
ME, kcal/kg	3226	3271	3315	3360
Ca	9.6	9.6	9.6	9.6
P	6.7	6.7	6.7	6.7
Non-phytate P	5.4	5.4	5.4	5.4
Na	1.7	1.7	1.7	1.7
Cl	2.2	2.2	2.2	2.2
Digestible amino acids, g/kg				
Arg	11.57	11.57	11.57	11.57
His	4.24	4.24	4.24	4.24
Ile	7.20	7.20	7.20	7.20
Leu	12.06	12.06	12.06	12.06
Lys	9.71	9.71	9.71	9.71
Met	2.18	2.18	2.18	2.18
Cys	2.35	2.35	2.35	2.35
Phe	7.99	7.99	7.99	7.99
Tyr	5.80	5.80	5.80	5.80
Thr	6.16	6.16	6.16	6.16
Trp	2.05	2.05	2.05	2.05
Val	7.50	7.50	7.50	7.50
TSAA	4.53	4.53	4.53	4.53
Phe + Tyr	13.79	13.79	13.79	13.79

1-4 Inclusion levels of lard 10, 20, 30, and 40 g /kg, respectively, in the experimental diets.

5 Vitamin A, 5484 IU; vitamin D3, 2643 ICU; vitamin E, 11 IU; menadione sodium bisulfate, 4.38 mg; riboflavin, 5.49 mg, d-pantothenic acid, 11 mg; niacin, 44.1 mg, choline chloride, 771 mg; vitamin B12, 13.2 µg; biotin, 55.2 µg; thiamine mononitrate, 2.2 mg; folic acid, 990 µg; pyridoxine hydrochloride, 3.3 mg.

6 Iodine, 1.11 mg; manganese, 66.06 mg; copper, 4.44 mg; iron, 44.1 mg; zinc, 44.1 mg; selenium, 300 µg.

### Sample collection

On d 21, all the birds were euthanized by CO₂ asphyxiation.

#### Expt. 1

Digesta from the distal half of the ileum (up to 5 cm proximal to the cecal junction) were collected by flushing with distilled water. The digesta were pooled from five birds per replicate to estimate BEL, AIAAD, and SIAAD. Additionally, sections of the duodenum (from the pyloric junction to the distal-most point of insertion of the duodenal mesentery), jejunum (from the distal-most point of insertion of the duodenal mesentery to the intersection with Meckel’s diverticulum), ileum (from the junction with Meckel’s diverticulum to the ileo-cecal junction), gizzard (emptied), and pancreas were collected from one bird per cage to measure the relative length and weight. Cecal content from one randomly selected bird per cage was collected and stored at −20°C to determine SCFA and pH.

#### Expt. 2

Digesta from the distal half of the ileum (up to 5 cm proximal to the cecal junction) was collected by flushing with distilled water to estimate SIAAD.

### Chemical analyses

The frozen ileal digesta were lyophilized and subsequently ground to pass through a 0.5 mm sieve. Dry matter was determined by drying the samples in a drying oven (VWR International, Radnor, PA, USA) at 100°C for 24 hours, using the AOAC method (Method 934.01). Titanium dioxide concentration in the sample was determined using the (Short et al., 1996) method. Nitrogen content was measured by the combustion method (AOAC method 990.03) in diets and digesta using a LECO N analyser (St Joseph, MI, USA). For amino acid analysis, samples were hydrolyzed in 6 N hydrochloric acid (Method 982.30) for 24 h at 110°C under a nitrogen atmosphere. Performic acid oxidation (Method 994.12) was used for Met and Cys prior to acid hydrolysis. Quantification was done using HPLC after post-column derivatization.

### Relative lengths and weights of digestive organs

To account for body weight differences, the weights and lengths of the duodenum, jejunum, and ileum, and the weights of the gizzard and pancreas were expressed relative to the body weight of the sampled birds.

### Ceca short-chain fatty acid content

Gas chromatography was used to analyze the SCFA content in the ceca as free fatty acids. Approximately 1 g of cecal content was diluted with 3 ml of distilled water (1:3 ratio) in 5 ml tubes. A 1.5 ml vortexed sample was centrifuged at 10,000 × g for about 10 min. Then, 1 ml supernatant was mixed with 0.2 ml of a 25% (w/v) metaphosphoric acid solution, and the samples were frozen overnight. After thawing the frozen samples and centrifuging at 10,000 × g for 10 min, the supernatant was combined with ethyl acetate in a 1:2 ratio, vortexed, and allowed to settle for 5 min. The top layer of the mixture was then analyzed by gas chromatography (Shimadzu GC-2010 plus, Shimadzu Corporation, Kyoto, Japan).

### pH of cecal content

Cecal content was thawed after being removed from the freezer at −20°C to determine the pH. Samples were weighed out and diluted 1:9 with distilled water, then stirred on a magnetic stirrer for 5 min to ensure uniform distribution. Then, a pH probe was placed into the solution, and the reading was determined with a digital-analog pH meter (Thermo Fisher Scientific, Waltham, MA, USA).

### Calculations and statistical analyses

#### Expt. 1 and Expt. 2

Apparent and standardized ileal digestibility were calculated as follows:

$$AIAAD\;=\left[1\;-\left(\frac{T_{i}}{T_{o}}\;\times \frac{AA_{o}}{AA_{i}}\right)\;\right]\times \;100;$$

$$SIAAD=\left[AIAAD+\left(\frac{BEL}{AA_{i}}\right)\right]\times \;100;$$

where.

AIAAD: apparent ileal AA digestibility (%).

AA_o_: the concentration of AA in digesta (%).

AA_i_: the concentration of AA in diets (%) for AIAAD calculation.

T_i_ and T_o_: concentrations of titanium dioxide (%) in the diets and digesta, respectively.

SIAAD: standardized ileal AA digestibility (%).

AA_i_: the concentration of AA in diets (g/kg) for SIAAD calculation.

BEL: basal endogenous AA losses (g/kg DMI).

The following equation was used to calculate the BEL at the terminal ileum as g AA flow per kg dry matter intake (**DMI**).

$$BEL=\;AA_{o}\;\times \frac{T_{i}}{T_{o}};$$

where:

BEL: - basal endogenous amino acid losses (g/kg DMI).

AA_o_: - concentration of amino acid (g/kg) in digesta.

T_i_ and T_o_: - concentrations of titanium dioxide (g/kg) in the diets and digesta, respectively.

### Statistical analysis

The data for Expt. 1 was analyzed using two-way ANOVA, through JMP software, and means were separated using Tukey HSD. The treatments were arranged in a 3 × 2 factorial with three feedstuffs and two fat types as factors. The data for Expt. 2 were analyzed using one-way ANOVA, and orthogonal polynomial contrasts were used to assess linear and quadratic effects across treatments.

## Results

The analyzed composition of test feedstuffs (corn, wheat, and wheat bran) shows that the crude protein and AA content of feedstuffs are within the expected range (corn < wheat < wheat bran, Table 4). Analysis of fiber revealed that both NDF and ADF were markedly higher in wheat bran compared to corn or wheat. The analyzed composition of experimental diets are presented in Table 5.

**Table 4:** Analyzed chemical composition (g/kg, as-fed) of the test feedstuffs for Expt. 1.

Items	Corn	Wheat	Wheat bran
Dry matter	877	880	893
Crude protein	93.4	104	147
Neutral detergent fiber	96.5	89.9	377
Acid detergent fiber	20.0	28.2	118
Hemicellulose	76.5	61.7	259
Indispensable amino acids			
Arg	4.20	5.40	10.3
His	2.70	2.80	4.30
Ile	3.30	3.90	4.70
Leu	11.2	7.30	8.90
Lys	3.00	3.80	6.80
Met	1.80	1.80	1.90
Phe	4.50	4.90	5.70
Thr	3.20	3.30	4.80
Trp	0.60	1.30	2.20
Val	4.30	5.00	7.00
Dispensable amino acids			
Ala	6.80	4.20	7.30
Asp	5.90	5.90	10.8
Cys	2.00	2.60	3.30
Glu	17.8	31.8	25.7
Gly	3.60	4.80	8.30
Pro	7.80	10.5	8.30
Ser	4.10	4.80	5.70
Tyr	2.80	2.50	3.60
Total	89.6	107	130

**Table 5:** Analyzed amino acid composition (g/kg) of experimental diets from d 16 to 21 for Expt.1.

Items	Corn		Wheat		Wheat bran	
Dry matter	890	896	900	899	910	910
Crude Protein	85.0	88.7	110	109	134	135
Indispensable amino acids						
Arg	4.10	4.30	5.60	5.50	9.60	9.70
His	2.70	2.70	2.70	2.70	4.00	4.10
Ile	3.20	3.10	3.70	3.70	4.30	4.30
Leu	10.9	10.7	7.00	6.90	8.10	8.10
Lys	3.00	3.10	3.70	3.70	6.30	6.30
Met	1.60	1.60	1.50	1.40	1.80	1.80
Phe	4.40	4.40	4.80	4.70	5.20	5.20
Thr	3.10	3.10	3.20	3.30	4.60	4.60
Val	4.40	4.40	4.90	4.70	6.40	6.40
Dispensable amino acids						
Ala	6.60	6.60	4.00	3.00	6.60	6.70
Asp	5.70	5.70	5.70	5.60	10.1	10.2
Cys	2.00	1.90	2.50	2.50	3.00	3.00
Glu	17.2	17.3	30.7	30.4	23.6	24.1
Gly	3.40	3.40	4.60	4.60	7.60	7.60
Pro	7.90	7.90	10.4	10.2	7.80	8.00
Ser	4.00	4.00	4.60	4.70	5.30	5.40
Tyr	3.30	3.20	3.00	3.00	3.50	3.50
Total	87.5	87.4	103	101	118	119

The fatty acid composition differed between soybean oil and lard (Table 6). Soybean oil contained a greater proportion of linolenic acid, and lard contained a greater proportion of oleic acid. Based on the calculated fatty acid classes, the major difference between the two fat types was the higher monounsaturated fatty acid (**MUFA**) in lard compared with soybean oil. Total saturated fatty acid (**SFA**), MUFA**,** and polyunsaturated fatty acid (**PUFA**) were further calculated to characterize the fat types.

**Table 6:** Analysis of fat profiles of the unsaturated and saturated fats used in the experiments.

	Soybean oil	Lard
C14:0	0.23	0.03
Myristoleic (9c-14:1)	ND^1^	ND^1^
C15:0	ND^1^	ND^1^
Palmitic (16:0)	12.30	14.33
Palmitoleic (9c-16:1)	0.22	0.12
Margaric (17:0)	0.36	0.07
10c-17:1	0.00	0.02
Stearic (18:0)	4.44	2.76
Elaidic (9t-18:1)	ND^1^	ND^1^
Oleic (9c-18:1)	16.97	26.25
Vaccenic (11c-18:1)	1.50	0.61
Linoleic (18:2n6)	44.80	52.39
Linolenic (18:3n3)	6.54	1.41
Arachidic (20:0)	0.29	0.57
Gonodic (20:1n9)	0.17	0.22
Total SFA^2^	17.62	17.76
Total MUFA^3^	18.86	27.22
Total PUFA^4^	51.34	53.80

1 Not detected.

2-4 Saturated fatty acids, monounsaturated fatty acids, polyunsaturated fatty acids.

### Expt. 1

There was a significant feedstuff × fat type (P < 0.05) for SIAAD of all indispensable AA except for Trp (Table 7). For wheat, the SIAAD tended to be greater (P < 0.10) for Arg, His, Leu, and Lys, when lard was used in the diet. For wheat bran, the fat type did not affect SIAAD, except for Lys, where soybean oil inclusion tended (P < 0.10) to increase digestibility. The SIAAD of corn was independent of the fat type used.

**Table 7:** Feedstuffs and fat type effect on standardized ileal amino acid digestibility (%) of indispensable amino acids in broiler chickens on d 21.

Feedstuff	Fat type	Arg	His	Ile	Leu	Lys	Met	Phe	Thr	Val	Trp
Main effect means of feedstuff											
Corn		88.2^a^	89.6^a^	85.9^a^	92.1^a^	77.0^a^	88.6^a^	88.9^a^	78.8^a^	86.2^a^	83.1^a^
Wheat		79.6^b^	83.5^b^	83.0^a^	84.3^b^	72.1^a^	83.3^a^	85.6^a^	76.1^a^	80.8^a^	90.0^a^
Wheat bran		61.8^c^	63.6^c^	55.3^b^	56.8^c^	46.5^b^	53.6^b^	58.4^b^	51.0^b^	55.3^b^	77.1^b^
Pooled SEM		1.51	1.42	1.79	1.49	2.51	1.73	1.52	2.29	1.71	2.29
Main effect means of fat type											
	Soybean oil	75.9	78.8	77.6	77.6	64.9	75.2	77.7	68.2	74.0	81.4
	Lard	77.2	79.0	74.7	77.8	65.8	75.1	77.4	69.1	74.2	85.3
Pooled SEM		1.27	1.20	1.51	1.26	2.12	1.46	1.28	1.93	1.44	1.93
Simple effect means											
Corn	Soybean oil	88.1^a^	89.8^a^	86.5^a^	92.2^a^	77.2^a^	89.2^a^	89.1^a^	79.4^a^	86.5^a^	79.4
Corn	Lard	88.3^a^	89.5^a^	85.3^a^	91.9^a^	76.8^a^	88.0^a^	88.6^a^	78.3^a^	85.8^a^	86.8
Wheat	Soybean oil	75.5^b^	80.2^b^	78.7^a^	80.5^b^	65.6^ab^	79.2^a^	82.2^a^	70.5^a^	76.9^a^	87.6
Wheat	Lard	83.9^ab^	86.8^ab^	87.3^a^	88.2^ab^	79.4^a^	87.4^a^	89.0^a^	81.8^a^	84.8^a^	92.4
Wheat bran	Soybean oil	64.1^c^	66.3^c^	59.0^b^	60.2^c^	51.7^bc^	57.3^b^	61.9^b^	54.8^b^	58.6^b^	77.3
Wheat bran	Lard	59.6^c^	60.9^c^	51.5^b^	53.4^c^	41.3^c^	50.0^b^	54.8^b^	47.1^b^	52.1^b^	76.8
Pooled SEM		2.13	2.01	2.53	2.11	3.55	2.44	2.15	3.24	2.42	3.24
					Probabilities						
Feedstuff		<0.001	<0.001	<0.001	<0.001	<0.001	<0.001	<0.001	<0.001	<0.001	0.003
Fat type		0.468	0.882	0.977	0.984	0.7522	0.957	0.869	0.763	0.905	0.212
Feedstuff × Fat type		0.021	0.027	0.016	0.009	0.009	0.017	0.015	0.0264	0.026	0.548

n = 6 replicate pens for simple effects; n = 18 replicate pens for the main effect of feedstuff, and n = 18 replicate pens for the main effect of fat type.

Means in the same column, within a group, but with different superscripts, are significantly different (P < 0.05).

There was a significant feedstuff × fat type (P < 0.05) for SIAAD of all dispensable AA except Cys, Glu, and Pro (Table 8). Fat type did not affect the SIAAD of corn. For wheat, SIAAD tended to be greater (P < 0.10) for Ala and Asp, with lard inclusion. For wheat bran, fat type did not affect SIAAD except for Asp, where soybean oil inclusion tended to increase (P < 0.10) digestibility.

**Table 8:** Feedstuffs and fat type effect on standardized ileal amino acid digestibility (%) of dispensable amino acids in broiler chickens on d 21.

Feedstuff	Fat type	Ala	Asp	Cys	Glu	Gly	Pro	Ser	Tyr
Main effect means of feedstuff									
Corn		90.6^a^	83.4^a^	85.4^a^	92.4^a^	80.7^a^	90.9^a^	86.4^a^	88.7^a^
Wheat		76.8^b^	75.2^b^	87.0^a^	93.3^a^	78.6^a^	92.7^a^	83.8^a^	85.2^a^
Wheat bran		55.7^c^	57.5^c^	65.9^b^	72.3^b^	58.0^b^	70.8^b^	59.5^b^	61.9^b^
Pooled SEM		1.65	1.95	1.48	0.95	1.82	1.05	1.63	1.48
Main effect means of fat type									
	Soybean oil	73.9	71.5	79.7	86.1	72.2	84.9	76.6	78.6
	Lard	74.9	72.6	79.1	85.8	72.8	84.7	76.6	78.6
Pooled SEM		1.39	1.64	1.25	0.80	1.53	0.89	1.37	1.24
Simple effect means									
Corn	Soybean oil	90.9^a^	83.8^a^	86.1	92.4	81.1^a^	91.0	86.7^a^	89.2^a^
Corn	Lard	90.4^a^	83.0^a^	84.6	92.3	80.3^a^	90.7	86.0^a^	88.2^a^
Wheat	Soybean oil	72.3^b^	69.9^bc^	84.4	91.8	74.5^a^	91.3	80.5^a^	81.5^a^
Wheat	Lard	81.4^ab^	80.5^ab^	89.6	94.8	82.8^a^	94.1	87.2^a^	88.8^a^
Wheat bran	Soybean oil	58.7^c^	60.6^cd^	68.4	74.2	60.8^b^	72.5	62.5^b^	65.1^b^
Wheat bran	Lard	52.8^c^	54.3^d^	63.3	70.3	55.2^b^	69.1	56.6^b^	58.7^b^
Pooled SEM		2.33	3.02	2.09	1.34	2.57	1.49	2.31	2.09
Probabilities									
Feedstuff		<0.001	<0.001	<0.001	<0.001	<0.001	<0.001	<0.001	<0.001
Fat type		0.642	0.664	0.771	0.769	0.785	0.833	0.977	0.931
Feedstuff × Fat type		0.014	0.019	0.071	0.063	0.045	0.154	0.046	0.013

n = 6 replicate pens for simple effects; n = 18 replicate pens for the main effect of feedstuff, and n = 18 replicate pens for the main effect of fat type.

Means in the same column, within a group, but with different superscripts, are significantly different (P < 0.05).

There were no significant interaction effects observed for relative lengths and weights of digestive organs (Table 9). The main effect of feedstuff was significant, with relative weights of duodenum, jejunum, ileum, and gizzard (P < 0.05) being greater for the birds that received wheat or wheat bran assay diet than for those that received corn assay diet, except for duodenum and gizzard weights. Birds that received the assay diet supplemented with lard had shorter jejunum (P = 0.037), and those that received lard tended (P = 0.093) to have greater relative pancreas weight. There was no significant feedstuff × fat type interaction or significant main effect of fat type on cecal content pH. Cecal content pH was significantly influenced by feedstuff (P < 0.01), with corn > wheat bran > wheat.

**Table 9:** Feedstuffs and fat type effects on the relative lengths and weights of intestine and digestive organs in broiler chickens on d 21.

			Duodenum		Jejunum		Ileum		Pancreas	Gizzard
Feedstuff	Fat type		Length	Weight	Length	Weight	Length	Weight	Weight	Weight
Main effect means of feedstuff										
Corn			0.192	0.069^b^	0.410	0.125^b^	0.399	0.095^b^	3.159	25.5^b^
Wheat			0.193	0.073^ab^	0.407	0.158^a^	0.400	0.127^a^	3.085	23.1^b^
Wheat bran			0.202	0.081^a^	0.402	0.173^a^	0.396	0.155^a^	3.176	30.5^a^
Pooled SEM			0.006	0.003	0.006	0.008	0.007	0.009	0.139	0.816
Main effect means of fat type										
	Soybean oil		0.192	0.073	0.414^a^	0.150	0.394	0.121	3.00	26.5
	Lard		0.199	0.076	0.398^b^	0.154	0.403	0.130	3.28	26.2
Pooled SEM			0.005	0.003	0.005	0.007	0.005	0.007	0.113	0.667
Simple effect means										
Corn	Soybean oil		0.189	0.069	0.408	0.119	0.403	0.096	3.00	25.7
Corn	Lard		0.194	0.069	0.411	0.131	0.395	0.095	3.32	25.3
Wheat	Soybean oil		0.184	0.067	0.422	0.155	0.394	0.108	2.99	23.6
Wheat	Lard		0.201	0.079	0.392	0.161	0.407	0.146	3.18	22.6
Wheat bran	Soybean oil		0.203	0.084	0.412	0.177	0.385	0.160	3.02	30.3
Wheat bran	Lard		0.201	0.079	0.392	0.169	0.407	0.150	3.33	30.7
Pooled SEM			0.009	0.004	0.009	0.012	0.010	0.013	0.196	1.155
Probabilities										
Feedstuff			0.414	0.019	0.681	0.009	0.886	0.003	0.886	<0.001
Fat type			0.354	0.539	0.037	0.705	0.279	0.402	0.093	0.747
Feedstuff × Fat type		0.579		0.120	0.151	0.685	0.290	0.151	0.931	0.855

Lengths and weights of the duodenum, jejunum, and ileum were calculated relative to the length of the small intestine.

The weights of the gizzard and pancreas were calculated relative to individual bird weight in g/kg.

Means in the same column, but with different superscripts, are significantly different (P < 0.05).

There was a significant feedstuff × fat type (P < 0.05) for cecal content isobutyrate and isovalerate concentration (Table 10). Whereas fat type did not affect the birds fed corn- or wheat-based assay diets, higher isobutyrate concentrations were observed with the use of soybean oil in the wheat bran diet. The feedstuff significantly influenced cecal SCFA concentrations; birds that received the corn assay diet had greater (P < 0.01) propionate and valerate concentrations, whereas those fed wheat bran had greater (P < 0.01) acetate and butyrate concentrations.

**Table 10:** Feedstuffs and fat type effects on the cecal content pH and short-chain fatty acid profile in broiler chickens on d 21.

Feedstuff	Fat type	pH	Acetate	Propionate	Isobutyrate	Butyrate	Isovalerate	Valerate	Caproate
Main effect means of feedstuff									
Corn		8.29^a^	13.5^b^	1.48^a^	0.12^a^	1.92^b^	0.08^a^	0.23^a^	0.14
Wheat		6.53^c^	11.0^b^	0.29^b^	0.00^c^	1.43^b^	0.00^b^	0.04^c^	0.28
Wheat bran		7.40^b^	20.0^a^	0.71^b^	0.08^b^	4.09^a^	0.06^a^	0.15^b^	0.26
Pooled SEM		0.225	1.55	0.147	0.009	0.438	0.007	0.016	0.067
Main effect means of fat type									
	Soybean oil	7.27	14.7	0.86	0.06	2.42	0.04	0.14	0.24
	Lard	7.55	15.0	0.79	0.08	2.54	0.06	0.15	0.21
Pooled SEM		0.184	1.25	0.118	0.007	0.352	0.006	0.013	0.053
Simple effect means									
Corn	Soybean oil	8.34	12.8	1.42	0.13^a^	1.87	0.09^a^	0.23	0.07
Corn	Lard	8.25	14.2	1.54	0.11^a^	1.97	0.08^ab^	0.24	0.22
Wheat	Soybean oil	6.20	12.5	0.39	0.00^b^	1.76	0.00^c^	0.05	0.30
Wheat	Lard	6.86	9.36	0.19	0.00^b^	1.10	0.01^c^	0.02	0.25
Wheat bran	Soybean oil	7.26	18.7	0.77	0.04^b^	3.64	0.03^bc^	0.13	0.34
Wheat bran	Lard	7.53	21.4	0.65	0.11^a^	4.54	0.09^a^	0.18	0.17
Pooled SEM		0.318	2.09	0.198	0.012	0.59	0.01	0.002	0.09
Probabilities									
Feedstuff		<0.001	<0.001	<0.001	<0.001	<0.001	<0.001	<0.001	0.335
Fat type		0.289	0.876	0.693	0.120	0.818	0.007	0.560	0.743
Feedstuff × Fat type		0.506	0.3705	0.717	0.009	0.444	0.004	0.288	0.245

Means in the same column, but with different superscripts, are significantly different (P < 0.05).

### Expt. 2

The SIAAD increased quadratically (P < 0.01) for all indispensable and dispensable AA as the level of lard in the assay diets increased (Table 11). Except for the L20 group, SIAAD increased stepwise as dietary lard inclusion increased, with Cys increasing the most and Pro the least.

**Table 11:** Effect of graded levels of lard on the SIAAD (%) of indispensable and dispensable amino acids in broiler chickens on d 21 for Expt. 2.

Indispensable amino acids									
Treatments	Arg	His	Ile	Leu	Lys	Met	Phe	Thr	Val
L10^1^	94.2	93.0	91.2	91.1	90.7	91.7	91.8	88.7	91.1
L20^2^	91.0	90.0	87.2	87.0	85.5	85.6	88.4	84.1	86.6
L30^3^	94.6	93.6	91.7	91.6	91.2	92.3	92.3	89.5	91.7
L40^4^	96.6	95.2	93.9	93.9	94.4	95.7	94.2	91.9	94.0
Pooled SEM	0.77	0.75	0.96	0.98	1.20	1.31	0.84	1.08	1.06
Probabilities	0.001	0.001	0.001	0.001	<0.01	<0.01	0.001	<0.01	0.001
Linear	0.005	0.005	0.007	0.007	0.004	0.004	0.007	0.004	0.007
Quadratic	0.003	0.006	0.004	0.003	0.002	0.001	0.005	0.004	0.004

Dispensable amino acids									
Treatments	Ala	Asp	Cys	Glu	Gly	Pro	Ser	Tyr	
L10^1^	90.3	90.7	80.0	93.7	88.8	92.9	91.1	90.9	
L20^2^	85.7	87.7	74.4	91.0	84.5	90.3	87.6	87.5	
L30^3^	91.7	91.1	83.0	94.1	89.6	93.5	92.0	91.6	
L40^4^	93.5	92.9	84.6	95.7	92.1	94.9	93.8	93.6	
Pooled SEM	1.13	0.76	1.63	0.69	1.08	0.68	0.84	0.86	
Probabilities	0.001	0.001	0.001	0.001	0.001	0.001	<0.01	0.001	
Linear	0.007	0.007	0.005	0.006	0.005	0.006	0.003	0.004	
Quadratic	0.004	0.005	0.037	0.004	0.005	0.008	0.004	0.004	

1-4 Inclusion levels of lard at 10, 20, 30, and 40 g /kg, respectively, in the assay diets.

## Discussion

The objectives of the current experiment were to investigate: 1) the interaction between fiber levels in feedstuff and fat used in the assay diets on SIAAD of the feedstuff, and 2) the effect of level of lard in the assay diets on SIAAD of SBM. We acknowledge that there are certain limitations in the experiments, including the observation that although we were interested in dietary fiber level in Expt. 1, the feedstuffs (corn, wheat, and wheat bran) are also different in other nutritional profiles apart from fiber content. In addition, the inclusion levels of the fat types differed across feedstuffs. This was done in an attempt to equalize the metabolizable energy content across diets, rather than using the same level of fat across diets, which would have resulted in different AME content for the assay diets.

### Expt. 1

In our previous work, we reported that fat types (soybean oil or tallow) had only minimal effects on the BEL and SIAAD of corn or SBM. Here, we investigated the interactivity of assay feedstuff fiber content and dietary fat type on SIAAD of the feedstuff. The objective is to evaluate the interactive effect of feedstuffs (corn, wheat, and wheat bran) differing in the NDF and ADF compositions, and the dietary fats (soybean oil or lard) that differ in their fatty acid composition, especially MUFA, on the SIAAD of the feedstuffs. We also examined the morphology of digestive organs, the SCFA profile of cecal content, and pH to identify clues that may explain the observed effects on SIAAD. We hypothesized that higher SIAAD estimates would be obtained with the assay diet containing soybean oil inclusion in corn. This may be attributed to the lower fiber content of corn compared with wheat or wheat bran, and to the fact that soybean oil is an unsaturated fat that is efficiently digested and absorbed. Several studies have reported the SIAAD of fiber feedstuffs (Adebiyi and Olukosi, 2015; Adedokun et al., 2015). The review conducted by (Jha and Mishra, 2021) reported a strong negative correlation between fiber and protein digestibility. Building on our previous study (Veeraganti et al., 2026), which examined the SIAAD of corn and SBM with different fat types, the current study aimed to determine the SIAAD of feedstuffs with differing fiber profiles, using fats with varying physical and chemical compositions. Because fat inclusion levels differed among assay feedstuffs, the effects of feedstuff and fat type cannot be completely separated. Although fat type comparisons were made within each feedstuff at equivalent fat inclusion levels, responses among feedstuffs may reflect differences in both feedstuff characteristics and fat inclusion level.

In the current study, a significant interaction effect (feedstuff × fat type) was observed for SIAAD, except for Trp, among indispensable AAs, and for Cys, Glu, and Pro among dispensable AAs. For assay diets with corn, there was no effect of fat type on SIAAD. This indicates that modifications to digesta due to fatty acid saturation did not constrain protein digestion in corn. It is known that corn has relatively lower levels of non-starch polysaccharides (**NSP**) (Knudsen, 2014). Lower NSP may result in a less viscous digesta environment. The emulsification or micelle-formation processes for fat types such as soybean oil or lard, unencumbered by the lower-viscosity digesta of corn diets, may have resulted in a lack of a fat-type effect on SIAAD in the feedstuff.

For assay diets containing wheat, SIAAD tended to be greater with the inclusion of lard for Arg, His, Leu, Lys, Ala, and Asp. It is known that soluble NSPs increase digesta viscosity, resulting in less efficient nutrient digestion and absorption (Ravindran and Amerah, 2009). The reason for the greater digestibility of some AA with lard inclusion is unclear. One possible explanation is that saturated fatty acids in lard may not increase mucin production or endogenous secretions relative to those from soybean oil due to its less efficient digestion, and, consequently, the lower endogenous amino acid losses may have led to the observed greater digestibility (Nieto et al., 2002).

For wheat bran assay diets, the SIAAD was not affected by the fat type, except for Lys and Asp, for which a greater SIAAD was observed with soybean oil inclusion. Wheat bran is rich in insoluble NSPs that make the digesta bulky in nature and affect nutrient absorption (Choct, 1997). The PUFA present in soybean oil enhance fluidity and emulsification compared to saturated fats, which help disperse digesta and enhance interactions between digestive enzymes and their substrates (Carlier et al., 1991). In addition, unsaturated fats may reduce digesta viscosity and increase the exposure of proteins to proteolytic enzymes, facilitating their digestion and absorption. This mechanism might overcome the bulkiness of the digesta caused by insoluble fibers in wheat bran (Sztupecki et al., 2023). The higher concentration of proteins in wheat bran is mostly in the aleurone layer, the outermost layer (Balandrán-Quintana et al., 2015), and might be sensitive to changes in enzyme activity enhanced by unsaturated fats. This may lead to greater SIAAD of Lys and Asp when soybean oil interacts with the fiber component. The main difference between the fat types in this study was the higher MUFA content in lard, mainly due to a higher oleic acid concentration. These compositional differences influence how dietary fat is digested and utilized by birds. In addition, the effect of fat type may depend on the characteristics of the feedstuff.

Trp among the indispensable AA, and Cys, Glu, and Pro among the dispensable AA were unaffected by the feedstuff × fat type interaction. However, their SIAAD was significantly higher in corn and wheat-based diets and lower in wheat-bran diets. This reflects differences in contrasting fiber composition among the feedstuffs used in this study, particularly the higher ADF and NDF content in wheat bran compared with corn and wheat. However, these responses may also reflect other compositional differences among feedstuffs. In our previous study (Veeraganti et al., 2026), both corn and soybean meal contained relatively lower fiber concentrations, which may have limited the extent to which fat type interacted with feedstuff to influence the SIAAD.

In our previous study, in addition to the SIAAD, we examined the histomorphology of small-intestinal sections and observed that diets and fat types influenced SIAAD, which was partly explained by changes in intestinal morphology. Therefore, in this study, we extended our approach to measuring the relative lengths and weights of small intestinal sections and digestive organs, as the assayed feedstuffs differ more markedly in fiber composition, which may alter gut morphology. Relative weights of small intestinal sections and the gizzard did not show a significant interaction effect; however, they increased significantly with the high dietary fiber in feedstuffs. Wheat bran had the highest fiber content among those used in the current experiment, and increased the relative weights of the duodenum, jejunum, ileum, and gizzard. This may be due to the higher mechanical and physiological activity associated with fiber. Wheat bran is less easily digested and requires greater intestinal motility and grinding activity. Earlier studies also reported higher relative weights of digestive organs with fiber inclusion (González-Alvarado et al., 2007; Amerah et al., 2009).

Consistent with current findings (Tejeda and Kim, 2021) reported that an increase in gizzard weight was observed with fiber inclusion, attributed to enhanced muscular grinding activity. While relative organ weights may be an adaptive response to high-fiber feedstuffs, they can also influence nutrient utilization. Fiber intake increases mucin production, contributing to BEL at the ileum (Onyango et al., 2008) which may partly explain the lower SIAAD with wheat and wheat bran diets compared with corn. The jejunum is the primary site of nutrient absorption in the small intestine; increasing its length proportionally increases surface area, thereby enhancing nutrient absorption, including amino acids. Soybean oil, rich in unsaturated fatty acids such as linoleic and linolenic acids, enhances intestinal development (Amer et al., 2021). In this study, birds that received diets with soybean oil had longer jejunum, whereas the SIAAD was unaffected by fat type across all AAs.

The inclusion of cecal content pH and organ morphology in the present study aimed to provide mechanistic insights into how variations in fiber composition of feedstuffs alter the gut environment, potentially influencing the endogenous amino acid flow and AA digestibility. Although these response criteria are not typically studied in SIAAD assays, evaluating physiological responses to differences in digestibility patterns driven by changes in microbial composition or intestinal development may offer valuable insights. Cecal content pH data also aligns with organ morphology results, with no interaction effect but a significant effect of feedstuff (wheat < wheat bran < corn). This has been attributed to active microbial fermentation in the cecum. In the study by (Jiménez-Moreno et al., 2009), it was reported that the inclusion of fiber feedstuffs reduced the pH of digesta, as also observed in the present study, even though the assayed feedstuffs differed in the experiments. The SIAAD in the present study was significantly higher with the corn diet, which is associated with its lower fiber content and comparatively reduced hindgut microbial fermentation. This is also supported by the higher cecal pH observed in birds that received corn than in those that received wheat or wheat bran, indicating reduced hindgut fermentation in the former.

The inclusion of fiber feedstuffs increased cecal microbiota and the number of SCFA-producing bacteria in the broiler gut (Liu et al., 2021; Ali et al., 2022). In the current study, cecal acetate and butyrate concentrations were significantly higher in wheat bran assay diets, indicating that dietary fiber promotes SCFA production. The higher butyrate concentration might also contribute to the observed increase in the relative weight of the intestine, as butyrate serves as a fuel for enterocyte development (Bedford and Gong, 2018). Concentrations of propionate and valerate were significantly higher in corn assay diets. Branched-chain fatty acids **(BCFA)**, isobutyrate, and isovalerate are indicative of protein fermentation rather than carbohydrate fermentation. In our previous study (Veeraganti et al., 2026) documenting the effect of fat type on BEL, we reported very low cecal BCFA concentrations in birds receiving the NFD. This is consistent with observations that BCFA are products of protein fermentation and are therefore expected to be low in birds fed diets without dietary nitrogen. In the current study, BCFA concentrations were generally low, but a significant interaction effect among treatments was observed. Fat type did not affect the BCFA concentrations in corn and wheat diets, whereas lard inclusion resulted in slightly higher BCFA levels than soybean oil in wheat bran diets. This might be due to the combined effects of high ADF and NDF in wheat bran and the saturated fatty acid profile of lard, which could alter nutrient utilization and substrate availability for microbial fermentation in the ceca, resulting in greater BCFA production. Although cecal fermentation occurs distal to the ileum, fermentation-derived metabolites such as SCFA may indirectly influence intestinal physiology (Qaisrani et al., 2015) ,including epithelial morphology and function, which can affect nutrient absorption capacity and thereby improve nutrient digestibility.

### Expt. 2

In our previous studies, we reported that fat type had only a marginal effect on BEL flow or SIAAD in corn and SBM (Veeraganti et al., 2026), using 15 g/kg soybean oil and 20 g/kg tallow in the assay diets. In the current study, we investigated whether the level of dietary saturated fat affects the SIAAD of feedstuff. In a study by (Skřivan et al., 2018), the ileal digestibility of fat was greater with lard inclusion at 60 g/kg than with the control or other dietary oils. However, no data are available on AA digestibility at different fat inclusion levels. We hypothesized that a stepwise increase in the fat inclusion level in the assay diet would produce a linear increase in the SIAAD of SBM until a plateau is reached. This is because the transit time of digesta in the gut increases with fat level, providing more time for nutrient absorption and thereby increasing nutrient digestibility (Mateos et al., 1982).

In the current study, as expected, the SIAAD of SBM increased linearly with stepwise increases in lard level from L10 to L40. However, the lower SIAAD for L20 resulted in a significant quadratic trend for all AA. The lower SIAAD in the L20 diet is unclear, but it is likely due to reduced titanium recovery in the digesta for this treatment. In a study, (Honda et al., 2009) using 3-10% tallow inclusion resulted in significantly higher crude protein digestibility of jejunal digesta, with the 10% inclusion, the highest level, showing the greatest effect in chickens. In the present study, Cys showed the greatest increase, and Pro the least. Cys, being sa sulfur-containing AA, contains a disulfide bond (Nte and Gunn, 2021), which may make it more sensitive and reactive to changes in intestinal dynamics influenced by dietary fat. In contrast, Pro has a cyclic structure, is high in mucin and endogenous secretions, and may not be affected by fat type, which is similar to observations in our previous study where the amount of BEL was unaffected by fat type (Veeraganti et al., 2026).

## Conclusions

Overall, both Expt. 1 and 2 assayed feedstuff characteristics, dietary fat type and level of fat inclusion influence the SIAAD estimates in broiler chickens. The interaction between feedstuff and fat type suggests that the composition of fat may modify AA digestibility. However, the mechanism underlying these effects needs further investigation. Therefore, estimation of SIAAD requires considering both feedstuff and fat characteristics to improve the precise feed formulation for broiler chickens.

## CRediT authorship contribution statement

**Sujitha Veeraganti:** Writing – original draft, Methodology, Investigation, Formal analysis, Data curation, Conceptualization. **Olanrewaju Ayinde:** Writing – review & editing, Methodology. **Oluyinka A. Olukosi:** Writing – review & editing, Supervision, Resources, Project administration, Investigation, Funding acquisition, Data curation, Conceptualization.

## Disclosures

The authors declare that they have no known competing financial interests or personal relationships that could have appeared to influence the work reported in this paper.
